# Evaluating carbapenem restriction practices at a private hospital in Manila, Philippines as a strategy for antimicrobial stewardship

**DOI:** 10.1186/s13690-019-0358-9

**Published:** 2019-07-04

**Authors:** Kaitlin F. Mitchell, Nasia Safdar, Cybele L. Abad

**Affiliations:** 1 Division of Infectious Diseases, Department of Medicine, University of Wisconsin-Madison, Madison, WI USA; 2 William S. Middleton Memorial Veterans Hospital, Madison, WI USA; 3 Infection Control Department, University of Wisconsin-Madison, Madison, WI USA; 4 Department of Medicine, Section of Infectious Diseases, The Medical City Hospital, Ortigas Ave, Pasig City, Philippines; 5 Present Address: Laboratory and Genomic Medicine, Department of Pathology and Immunology, Washington University School of Medicine in St. Louis, St. Louis, MO USA

**Keywords:** Antimicrobial stewardship, Philippines, Carbapenem, Empiric and definitive prescriptions

## Abstract

**Background:**

Hospital antimicrobial stewardship programs are especially critical in countries such as the Philippines, where antibiotic resistant infections are highly prevalent. At the study institution in Manila, Philippines, a Prior Approval for Restricted Antimicrobials (PARA) is required for non-infectious disease specialists to prescribe certain antimicrobials, including carbapenems. PARA request forms include specification of empiric or definitive therapy based on diagnostic tests. Recommended duration of therapy is typically 3 days for empiric use and 7 days for definitive, with possible extension upon specialist approval.

**Methods:**

The study took place at an 800-bed tertiary hospital. We performed a retrospective review of patient medical records and laboratory reports dating from January 1 to December 31, 2016. Information related to patient demographics, carbapenem prescription, laboratory diagnosis, and therapy were compiled. Carbapenem prescriptions were classified as ‘adherent’ or ‘non-adherent’ according to clinical guidelines related to infection diagnosis, treatment duration, and de-escalation.

**Results:**

Of the 185 patients on carbapenem therapy, Prescriptions of carbapenems were either definitive (*n* = 56), empiric (*n* = 127), or prophylactic (*n* = 2) as defined by the ordering provider. 69 out of 185 (37%) prescriptions were deemed non-adherent to guidelines, despite receiving approvals for their respective requests. Of these, 72% were non-adherent due to failure to de-escalate the carbapenem and 28% were non-adherent due to an incomplete course of therapy.

**Conclusion:**

Despite initial PARA approval for carbapenem therapy, 37% of prescriptions were non-guideline-adherent, highlighting the ongoing challenges in implementing this type of stewardship strategy. In order to increase the effectiveness of PARA, additional approaches may be warranted, including the application of strict policies which reinforce follow-up of available culture results, justification of therapy extension, or referral to an infectious disease specialist.

**Electronic supplementary material:**

The online version of this article (10.1186/s13690-019-0358-9) contains supplementary material, which is available to authorized users.

## Background

Antibiotic resistance is disproportionately abundant in low- and middle- income countries such as the Philippines. Multiple factors contribute to this: patients can often obtain antibiotics without a prescription, many healthcare providers write unnecessary prescriptions, and infectious disease surveillance systems may be inadequate [1–3]. Studies in the Philippines have identified issues such as self-prescription, use of antibiotics for unconfirmed bacterial infections, and dispensing of incomplete antibiotic courses by both community pharmacies and clinics [4, 5]. Given these factors, patients are more likely to develop antibiotic resistance to these readily-available drugs and require more advanced antibiotics. Healthcare facilities are the source of such antibiotics for many individuals, and have the ability to provide drugs of ‘last resort’ that are not typically available elsewhere [3]. Inappropriate use of these antimicrobial agents is a significant contributor to the development of multi-drug resistant organisms (MDROs) and untreatable infections [6].

Similar to other healthcare facilities in the region, our hospital has high rates of MDROs. For example, data collected from 2016 revealed high rates of *Pseudomonas aeruginosa* with resistance to last-resort antibiotics: among respiratory isolates from the intensive care unit, 31 and 37% were resistant to piperacillin-tazobactam and carbapenems, respectively (unpublished data).

To help preserve the effectiveness of existing antimicrobials, many hospitals have implemented antimicrobial stewardship programs (ASPs). The primary goal of any ASP is to optimize clinical outcomes while minimizing the unintended consequences of antimicrobial utilization, which include the development of resistance and toxicity [7, 8]. These programs can be especially critical in reducing unnecessary carbapenem utilization [9, 10]. One evidence-based strategy for achieving this is formulary restriction with prior authorization for select agents [7, 8]. Formulary restriction can be accomplished by requiring prior approval for use by an infectious disease (ID) specialist. While this method can be effective, it requires highly-trained personnel [11].

The present study examines one component of the ASP at our large, tertiary hospital located in Manila, Philippines: the Prior Approval for Restricted Antimicrobials (PARA). The role of formulary restriction with prior authorization for select agents has not been evaluated at this institution. We undertook a retrospective study to evaluate the effect of this strategy on carbapenem prescribing.

## Methods

### Study setting and population

This retrospective study was conducted at The Medical City, a private, 800-bed, tertiary hospital in Manila, Philippines. This hospital has had an ASP in place since 2002. The Prior Approval for Restricted Antimicrobials (PARA) is an integral part of this program that requires approval from an infectious disease (ID) specialist prior to the prescription of restricted antibiotics, including carbapenems (meropenem, ertapenem, and imipenem) (Additional file 1).

We evaluated patients with a request for carbapenem therapy through the PARA process between January 1 and December 31, 2016.

### Description of prior approval for restricted antimicrobials (PARA)

PARAs can be requested by phone 24 h a day, 7 days a week, and are granted by a clinical pharmacist after approval by the ID specialist. Following both clinical and institutional guidelines, *definitive* prescriptions of 7 days are recommended for infections with a laboratory-confirmed diagnosis while *empiric* prescriptions for 3 days are suggested for infections prior to diagnosis [12–14]. *Prophylactic* prescriptions are those given for 1–2 doses, prior to device insertion or surgery (Additional file 2). Longer durations of therapy must be approved by a PARA extension, otherwise the pharmacist releases an automatic stop order. Once laboratory data is available for empiric prescriptions, the carbapenem should be de-escalated if warranted, though this is not specifically indicated in the hospital’s policy.

### Inclusion and exclusion criteria

Inclusion criteria were either the approval or disapproval for any carbapenem prescription within the study period. For patients with multiple PARA requests, only the first approved request for carbapenem use was included. Subsequent requests were included in the analysis only if they were for a different indication (i.e. different infection site or different carbapenem requested). Patients were excluded from the study if their carbapenem was prescribed by an ID specialist, as these physicians do not need a PARA request to prescribe carbapenems. Patients were also excluded if the PARA request was for a restricted antibiotic other than a carbapenem.

### Data collection and analysis

A list of patients with PARA requests was compiled by the hospital’s infection control department. Patient record numbers from this list were used to manually search for medical records from the hospital’s online database (Medical Information Data Analysis System, or MIDAS). Electronic laboratory records were queried to obtain culture results and antibiotic susceptibility data.

Data collected included: patient demographics, co-morbidities, duration of hospital stay, antimicrobial dosage and duration of therapy, culture data (isolated pathogen, infection site, and susceptibility data), and patient outcomes (survival or death at end of hospital stay). MDROs were defined as organisms with reported resistance to one or more antibiotic classes. Carbapenem prescriptions were categorized as guideline-adherent or non-adherent, with prescription adherence defined as follows: 1) carbapenems de-escalated based on laboratory data, or 2) completion of the antibiotic course. In addition, antibiotic courses exceeding the recommended duration for empiric and definitive prescriptions were considered adherent if there was an ID specialist assigned to the patient case to approve the PARA extension.

### Statement of ethical compliance

The study was approved by the Institutional Review Board (IRB) at the site in the Philippines. The study was considered exempt from review by the University of Wisconsin-Madison IRB, as it qualified as a quality improvement study.

## Results

275 patient cases were identified from the initial database of PARA requests in 2016. After applying exclusion criteria, 185 cases remained for analysis. Prescriptions of carbapenems were either definitive (*n* = 56), empiric (*n* = 127), or prophylactic (*n* = 2) as defined by the ordering provider. For the two cases with prophylactic carbapenems, prescriptions were requested during the placement of a surgical device, because of history of prior drug resistance. The median patient age was 75.5 years (range 23 to 98), 45% of cases were male, and common comorbidities included type 2 diabetes and hypertension. The most common site of infection was the respiratory tract (77.3% of cases) (Table 1).

**Table 1:** Characteristics of patients with a Prior Approval for Restricted Antimicrobials (PARA) request between January 1 and December 31, 2016. Manila, Philippines. “Evaluating carbapenem restriction practices at a private hospital in Manila, Philippines as a strategy for antimicrobial stewardship”

	Total n (%)^a^	Definitive n (%)	Empiric n (%)	Prophylactic n (%)
Group size (N)	185	56	127	2
Age (median years)	75.5	78.4	72.7	75.0
Gender (male)	83 (45%)	30 (54%)	52 (41%)	1 (50%)
Total # comorbidities of interest	268	83	183	2
Average # comorbidities per patient	1.45	1.48	1.44	1.00
Chronic Obstructive Pulmonary Disorder	25	8	16	1
Hypertension	102	36	66	0
Diabetes Mellitus	66	23	43	0
Malignancy (any, active)	51	9	41	1
Hemodialysis	25	6	19	0
None	28	7	20	1
Outcome				
Length of hospital days (median days)	14.0	14.5	14.0	15.0
Mortality	42 (23%)	10 (18%)	32 (25%)	0 (0%)
Cases with same recurrent infection within 30 days of discharge	14 (8%)	2 (4%)	12 (9%)	0 (0%)
Duration of therapy (median days)	6.5	7.0	5.0	11.0
Site of infection for PARA request				
Respiratory	145	30	113	2
Blood	18	8	10	0
Gastrointestinal	1	1	0	0
Genitourinary	39	24	15	0
Wound/surgical site	2	1	1	0

^a^Percentage within the column

Organism identification data was collected from the hospital’s microbiology laboratory reports. 276 clinical isolates were reported from the 185 patients. Hospital acquired enteric and Gram-negative organisms that are commonly associated with carbapenem resistance accounted for 56.2% of diagnoses (Table 2). 229 of 276 (83.0%) of the isolates had susceptibility testing reported, and 101 of these (44.0%) were MDROs.

**Table 2:** Bacterial species identified as reported by the microbiology laboratory (*n* = 276). 2016, Manila, Philippines. “Evaluating carbapenem restriction practices at a private hospital in Manila, Philippines as a strategy for antimicrobial stewardship”

Organism	Number of isolates, *N* = 276 n (%)
*Klebsiella pneumoniae*	53 (19.2)
*Escherichia coli*	42 (15.2)
*Acinetobacter baumanii*	20 (7.2)
*Pseudomonas aeruginosa*	15 (5.4)
*Enterobacter cloacae*	13 (4.7)
*Citrobacter* spp.^a^	6 (2.2)
*Klebsiella oxytoca*	2 (0.7)
*Proteus mirabilis*	2 (0.7)
*Serratia marcescens*	2 (0.7)
Negative	36 (13.0)
Other	85 (30.8)
*Candida albicans*	38 (44.7)^b^
*Staphylococcus aureus*	15 (17.6)
*Stenotrophomonas maltophila*	10 (11.8)
*Enterococcus faecalis*	3 (3.5)
*Achromobacter xylosoxidans*	2 (2.4)
*Aeromonas hydrophila*	2 (2.4)
*Enterococcus faecium*	2 (2.4)
*Haemophilus influenzae*	2 (2.4)
Singletons	11 (12.9)

^a^Included *C.freundii* and *C. koseri*

^b^Percentage from ‘Other’ group, containing organisms not typically treated with carbapenems

Using the guidelines defined above, 69 out of 185 (37%) prescriptions were deemed non-adherent, despite receiving approvals for their respective PARA requests. Of these, 50 (72%) were non-adherent as there was no de-escalation of the carbapenem and 19 (28%) were due to an incomplete course of therapy. These values were also calculated within groups for empiric, definitive, and prophylactic prescriptions and are shown in Table 3.

**Table 3:** Number of carbapenem prescriptions following Prior Approval for Restricted Antimicrobials (PARA) by adherence to clinical guidelines and by the reason for non-adherence. 2016, Manila, Philippines. “Evaluating carbapenem restriction practices at a private hospital in Manila, Philippines as a strategy for antimicrobial stewardship”

	Total n (%)	Definitive n (%)	Empiric n (%)	Prophylactic n (%)
Total cases	185	56	127	2
Guideline-adherent	116 (63%)	43 (77%)	71 (56%)	2 (100%)
Non-guideline-adherent	69 (37%)	13 (23%)	56 (44%)	0 (0%)
Total non-guideline-adherent cases	69	13	56	–
No de-escalation	50 (72%)	5 (38%)	45 (80%)	–
Incomplete course	19 (28%)	8 (62%)	11 (20%)	–

Several notable details related to these non-adherent prescription classifications were encountered in the patient records. First, failure to de-escalate from carbapenems was sometimes observed in the absence of a laboratory result (in contrast to a result of “No organism” or “Other organism”). Second, an incomplete course of antibiotics was considered non-adherent only for reasons within the control of the provider. These reasons included 1) the discharge of a patient prior to full carbapenem course (without indication for antibiotic continuation as an outpatient), 2) patient or family member’s refusal to finish the carbapenem course, sometimes due to financial concerns, 3) or note of clinical improvement/resolution with the carbapenem that justified discontinuation of therapy, or 4) no reason was stated in the charts.

## Discussion

Our study describes the PARA program, the approval process for restricted antibiotics at a hospital in Manila, Philippines. Since its inception in 2002, the program has not been evaluated in terms of subsequent antibiotic prescriptions following the approval process. In our study, we found the majority (63%) of prescriptions using PARA were guideline-adherent.

It is challenging, especially in resource-limited environments, to maintain an ASP with coordination of physicians, the laboratory, infection control, and clinical pharmacists [1, 15]. At this institution, we found that carbapenem prescriptions granted within the PARA program were more often adherent to clinical practice guidelines than not. In addition, many patients who were discharged prior to the end of their prescriptions were provided instruction for outpatient care, which often involved intravenous administration. Despite these program strengths, the proportion of prescriptions that deviated from clinical guidelines (37%) underscores some gaps of the current strategy.

The PARA program does not mandate adjustment of therapy upon the availability of microbiological culture results. We found this was particularly an issue for empiric prescriptions, which are meant to provide reasonable antibiotic therapy until culture data are available, usually within 48 to 72 h [16]. Even when laboratory results indicated a negative culture or an irrelevant organism, carbapenems were not always discontinued or de-escalated to a narrow-spectrum antibiotic. This scenario, which occurred for 44.5% of PARA prescriptions, represents a clear point where carbapenems should be de-escalated. The decisions regarding therapy in these cases were often left to providers rather than referred to an ID specialist. Full utilization of a microbiology laboratory’s testing capability is critical to the success of an ASP, as has been demonstrated in other healthcare settings with limited-resources [15, 17, 18].

Lack of clinical confidence has been cited as a barrier to successful de-escalation [19] – if an antibiotic is noticeably improving a patient’s symptoms, there may be reluctance to change. Previous studies have shown that de-escalation strategies reduce antimicrobial resistance levels and patient mortality without compromising patient safety, but also acknowledge difficulties in implementation [19–21]. As our study corroborates, de-escalation policies should be supplemented by implementation support; providing physicians with additional support from ID specialists or clinical pharmacists at these key decision-making moments, perhaps through direct audit and feedback, or requiring follow up consults by phone or in-person, are essential. Asking providers to describe their prescribing choices at set time points has been shown to increase accountability [22], and could be a useful part of these consults. In addition, stewardship interventions led by ID specialists have led to reduced carbapenem utilization and subsequent reduction in antibiotic resistance rates [9, 10].

Another reason for non-adherent prescriptions was premature shortening of antibiotic courses, which is discouraged in current guidelines [23, 24]. Both the definitive and empiric prescription groups in our study included patients given incomplete courses of carbapenems. Our analysis excluded cases with therapy that was shortened for reasons beyond the control of a provider (e.g. patient death). However, other reasons for non-adherent carbapenem shortening may be preventable. Patients who need ongoing carbapenem therapy should enter a formal outpatient antibiotic treatment program. In several cases, patients’ financial concerns were noted as the reason for incomplete antibiotic courses, representing an important area of future work in stewardship programs.

In our cohort, *K. pneumoniae* and *E. coli* were the most commonly isolated organisms at 19.2 and 15.2%, respectively. This distribution of pathogens is unsurprising and mirrors national data: in a 2017 report of 76,892 clinical isolates from the national Antimicrobial Resistance Surveillance Program [25], *K. pneumoniae* (16.4%) and *E. coli* (11.6%) accounted for the top 2 isolated organisms as well.

Our study had several limitations. Review of patient charts involved reading numerous scanned pages with handwritten text. Since these were non-searchable for key words, certain notes of interest may have been missed. Furthermore, many patient records did not contain any laboratory reports for the infection of interest. Based on the information available in the charts at this institution, the underlying reasons for absent cultures were unclear – the test may have been ordered but not carried out because of the inability of the patient to provide a sample; in most cases of hospital-acquired pneumonia, for example, the patient is unable to expectorate sputum. We were unable to capture carbapenem prescriptions made by ID physicians which can provide useful information regarding overall carbapenem use within the hospital. Also, the use of ancillary laboratory diagnostics such as procalcitonin, which may have been used to help determine treatment length, was not captured in this study. Finally, this was a single-center analysis conducted at a private hospital. This facility has an established framework for infection control and antimicrobial stewardship, so findings for this specific program may not be applicable to facilities without an ASP currently in place.

## Conclusions

The global challenge presented by antimicrobial resistance necessitates the establishment of ASPs to curtail inappropriate use of antibiotics in healthcare facilities. Our study highlights some of the issues that may be faced by institutions in resource-limited settings when implementing these policies. Opportunities for intervention include greater utilization of available laboratory resources and mandating the adjustment of therapy based on test results. Increasing decision-making support for providers on optimizing carbapenem use may also be of benefit.

## Additional files

Additional file 1: Algorithm for PARA (Prior Approval for Restricted Antimicrobials). (DOCX 145 kb)

Additional file 2: Summary of empiric antibiotic therapy guidelines used at the study site for developing the PARA program. Guidelines were derived from national and local guidelines in the Philippines for empiric antibiotic therapy [11, 12] and institutional antibiogram data (data not shown). (DOCX 18 kb)

## Data Availability

The datasets used and/or analyzed during the current study are available from the corresponding author on reasonable request.

## Abbreviations

ASP: Antimicrobial stewardship program
ID: Infectious Disease
MDRO: Multi Drug Resistant Organism
PARA: Prior Approval for Restricted Antimicrobials
