# Supplementary material for: Evaluating carbapenem restriction practices at a private hospital in Manila, Philippines as a strategy for antimicrobial stewardship
Source: Arch Public Health. 2019 Jul 4;77:31. doi: 10.1186/s13690-019-0358-9 (PMC6610803; doi:10.1186/s13690-019-0358-9)
Supplement: Supplementary file 1 — Algorithm for PARA (Prior Approval for Restricted Antimicrobials). (DOCX 145 kb) [file 13690_2019_358_MOESM1_ESM.docx]

**Appendix 1.** Algorithm for PARA (Prior Approval for Restricted Antimicrobials)
