# Supplementary material for: Evaluating carbapenem restriction practices at a private hospital in Manila, Philippines as a strategy for antimicrobial stewardship
Source: Arch Public Health. 2019 Jul 4;77:31. doi: 10.1186/s13690-019-0358-9 (PMC6610803; doi:10.1186/s13690-019-0358-9)
Supplement: Supplementary file 2 — Summary of empiric antibiotic therapy guidelines used at the study site for developing the PARA program. Guidelines were derived from national and local guidelines in the Philippines for empiric antibiotic therapy [11, 12] and institutional antibiogram data (data not shown). (DOCX 18 kb) [file 13690_2019_358_MOESM2_ESM.docx]

**Appendix 2.** Summary of empiric antibiotic therapy guidelines used at the study site for developing the PARA program. Guidelines were derived from national and local guidelines in the Philippines for empiric antibiotic therapy^11,12^ and institutional antibiogram data (data not shown).

**1. Urine isolates**

- **Acute cystitis**: nitrofurantoin or fosfomycin (3g single dose)
- **Acute uncomplicated pyelonephritis**:
  - ceftriaxone
- **In-patients (hospital-acquired):**
  - without risk factors for ESBL-producing organisms: piperacillin-tazobactam OR amikacin
  - with risk factors for ESBL-producing organisms: ertapenem OR amikacin

**Risk Factors for ESBL-producing organisms, Urine**

- Previous antibiotic use- cephalosporins, fluoroquinolones
- Previous international travel
- Diabetes Mellitus
- Age >60
- Female sex
- Recent UTI
- Structural or anatomic abnormality
- History of fluoroquinolone intake for UTI in preceding 3 months
- Recent urinary tract surgery or instrumentation

**2. Respiratory isolates**

- **Community-acquired pneumonia – MODERATE RISK**
  - ampicillin-sulbactam 1.5gm IV every 6 hours OR
  - cefuroxime 1.5gm IV every 8 hours OR
  - ceftriaxone 2gm IV once a day

PLUS

- - azithromycin 500mg PO once a day OR
  - clarithromycin 500mg PO twice a day OR
  - levofloxacin 750mg PO once a day
- **Community-acquired pneumonia – HIGH RISK, without risk factors for *P. aeruginosa***
  - ceftriaxone 2gm IV once a day OR
  - ertapenem 1gm IV once a day

PLUS

- - azithromycin dehydrate 500mg IV once a day OR
  - levofloxacin 750 mg IV once a day
- **Community-acquired pneumonia – HIGH RISK, WITH risk factors for *P. aeruginosa***
  - piperacillin-tazobactam 4.5gm IV every 6h OR
  - cefepime 2gm IV every 8-12h OR, PLUS
  - azithromycin 500mg IV once a day PLUS amikacin 15mg/kg IV once a day
- **Community-acquired pneumonia – HIGH RISK, with suspicion of MRSA, ADD:**
  - vancomycin  25-30 mg/kg IV loading dose then 15-20 mg/kg every-8-12 hours OR
  - linezolid 600mg IV every 12 hours OR
  - clindamycin 600mg IV every 8 hours
- **HAP/VAP – inpatient, no risk factors for MDR organisms (e.g. *A. baumannii*)**
  - Piperacillin-tazobactam OR cefepime OR meropenem ±
  - Amikacin OR levofloxacin
- **Pneumonia other than CAP – inpatient, with risk factors for MDROs (e.g. *A. baumannii*)**
  - Immediate referral to ID recommended
- **Pneumonia other than CAP – inpatient, non-ICU, with risk factors for MRSA or aspiration**
  - Add clindamycin
- **Pneumonia other than CAP – inpatient, ICU, with risk factors for MRSA**
  - Add vancomycin OR linezolid

**Risk Factors, Respiratory Isolates**

MRSA Pneumonia

- Recent hospitalization
- Recent intake of antibiotics
- Surgery
- Chronic illness
- Those who have central lines
- Patients undergoing chronic hemodialysis
- Exposure to MRSA colonized person
- Athletes (wrestlers, football players)
- IVDU
- Those in nursing home, jails

Risk factors for MDROs such as MDR-*Pseudomonas* or *Acinetobacter*

For HAP/VAP:

- Receipt of antibiotics within the preceding 90 days
- Current hospitalization of ≥5 days
- High frequency of antibiotic resistance in the community or in the specific hospital unit
- Immunosuppressive disease and/or therapy

For HCAP:

- Hospitalization for ≥2 days during the preceding 90 days
- Severe illness
- Antibiotic therapy in the past six months
- Poor functional status as defined by activities of daily living score
- Immune suppression

Risk factors for *Pseudomonas aeruginosa*:

- Immunocompromised state (HIV, post-transplantation, neutropenia, on immunosuppressive and immunomodulatory agents)
- Recent antibiotic use
- Liver cirrhosis
- Structural lung abnormalities (bronchiectasis, cystic fibrosis)
- Repeated exacerbations of COPD requiring glucocorticoids and frequent antibiotic use

**3. Wound isolates**

- **Skin and Soft Tissue Infections**
  - Caveat: The vast majority of non-purulent SSTI (i.e. no microbiologic data available) is due to Streptococci
- **Community acquired, localized, without signs of sepsis:**
  - Clindamycin OR
  - Doxycycline OR
  - Co-trimoxazole (no good Streptococcal coverage)
- **Nosocomial and/or with signs of sepsis:**
  - Vancomycin OR Linezolid PLUS piperacillin-tazobactam
